# Nocturnal Blood Pressure Pattern Affects Left Ventricular Remodeling and Late Gadolinium Enhancement in Patients with Hypertension and Left Ventricular Hypertrophy

**DOI:** 10.1371/journal.pone.0067825

**Published:** 2013-06-26

**Authors:** Hajime Yokota, Yasuko Imai, Yusuke Tsuboko, Aya M. Tokumaru, Hajime Fujimoto, Kazumasa Harada

**Affiliations:** 1 Department of Cardiology, Tokyo Metropolitan Geriatric Hospital, Itabashi, Tokyo, Japan; 2 Department of Radiology, Tokyo Metropolitan Geriatric Hospital, Itabashi, Tokyo, Japan; University of Minnesota, United States of America

## Abstract

**Background:**

Left ventricular hypertrophy (LVH) is an independent predictor of cardiac mortality, regardless of its etiology. Previous studies have shown that high nocturnal blood pressure (BP) affects LV geometry in hypertensive patients. It has been suggested that continuous pressure overload affects the development of LVH, but it is unknown whether persistent pressure influences myocardial fibrosis or whether the etiology of LVH is associated with myocardial fibrosis. Comprehensive cardiac magnetic resonance (CMR) including the late gadolinium enhancement (LGE) technique can evaluate both the severity of changes in LV geometry and myocardial fibrosis. We tested the hypothesis that the nocturnal non-dipper BP pattern causes LV remodeling and fibrosis in patients with hypertension and LVH.

**Methods:**

Forty-seven hypertensive patients with LVH evaluated by echocardiography (29 men, age 73.0±10.4 years) were examined by comprehensive CMR and 24-h ambulatory blood pressure monitoring (ABPM).

**Results and Conclusions:**

Among the 47 patients, twenty-four had nocturnal non-dipper BP patterns. Patients with nocturnal non-dipper BP patterns had larger LV masses and scar volumes independent of etiologies than those in patients with dipper BP patterns (p = 0.035 and p = 0.015, respectively). There was no significant difference in mean 24-h systolic BP between patients with and without nocturnal dipper BP patterns (p = 0.367). Among hypertensive patients with LVH, the nocturnal non-dipper blood pressure pattern is associated with both LV remodeling and myocardial fibrosis independent of LVH etiology.

## Introduction

Left ventricular hypertrophy (LVH) increases morbidity and mortality in patients with hypertension [1] [2] [3]. Elevated blood pressure (BP) contributes to wall thickening and functional changes [4]. These changes cause systolic and diastolic dysfunction and their clinical manifestations include arrhythmia and symptomatic heart failure. A previous echocardiographic study revealed that nocturnal high BP affects LV mass and function [5]. According to a recent study [6], nocturnal BP is the most significant prognostic maker of cardiovascular morbidity and mortality. It has been suggested that continuous pressure overload affects the development of LVH, but it is unknown whether persistent pressure influences myocardial fibrosis or whether the etiology of LVH is associated with myocardial fibrosis. Comprehensive cardiac magnetic resonance (CMR) including the late gadolinium enhancement (LGE) technique can evaluate both the severity of changes in LV function, geometry, and myocardial fibrosis. Comprehensive CMR can also provide information about the causes of LVH [7] and cardiac mortality of LVH of any cause [8] [9] [10]. Thus, the aim of our study was to investigate the effect of nocturnal BP on the myocardium in hypertensive patients with LVH by means of comprehensive CMR.

## Methods

### Patient Population

Forty-seven hypertensive patients with LVH evaluated by echocardiography (29 men, 18 women; mean age 73.0±10.4 years) were prospectively examined by comprehensive CMR and 24-h ambulatory blood pressure monitoring (ABPM) between May 2010 and May 2012. Hypertensive patients were defined either by taking antihypertensive agents or with systolic BP≥140 mm Hg and/or diastolic BP≥90 mm Hg in the clinic. The diagnosis of LVH was based on the demonstration before comprehensive CMR by 2-dimensional echocardiogram of a hypertrophied left ventricle wall thickness >12 mm. Patients who had myocardial infarction, amyloidosis, aortic stenosis (AS), or contraindications to CMR imaging were not included. We also did not include patients with previous septal ablation or myectomy. Diagnosis of AS was based on a Doppler echocardiographic demonstration of a peak aortic valve pressure gradient >36 mm Hg and peak transvalvular velocity >3m/s [9]. Twenty-four-h ABPM was performed using TM 243 (A & D Medical Co. Ltd, Tokyo Japan). BP recordings were obtained automatically every 30 min throughout a 24-h period. The nocturnal dipper BP pattern was defined as a mean nocturnal systolic BP decline grater than 10% relative to mean daytime systolic BP. The nocturnal non-dipper BP pattern was defined as a mean nocturnal systolic BP decline less than 10% and riser relative to mean daytime systolic BP. Estimated glomerular filtration rate (eGFR) was estimated using the equation for Japanese patients by the Japanese Society of Nephrology Chronic Kidney Disease Initiatives (JSN-CKDI) [11]. This study was inducted in accordance with the Declaration of Helsinki. This study protocol was approved by the Ethics Committee at Tokyo Metropolitan Geriatric hospital and all subjects provided written inform consent.

### Imaging Protocols

All images were acquired on a 1.5-Tesla whole-body scanner (Signa HD xt 1.5 ver 15, GE Healthcare, Milwaukee, WI). Cine images were acquired using a steady-state free precession sequence (SSFP, TR 3.8, TE 1.6, FA 45°, slice thickness 10mm, slice gap 0). LGE images (segmented k-space inversion recovery sequence, TR Auto, TE MinF, TI 130–260, slice thickness 9mm, slice gap 0) were acquired throughout the entire LV starting at 5 min, following administration of 10 mmol meglumine gadoterate (Gd-DOTA, MAGNESCOPE®, Guerbet, Japan). The inversion time set to null the signal of normal myocardium after Gd-DOTA administration was adjusted during the course of the scan as necessary.

### CMR Analysis

Images were analyzed using Advantage Workstation (AW Volume Share 2 Version 4.4, GE Healthcare, Milwaukee, WI). Manual tracing and adjustment of endocardial and epicardial borders from short-axis images was performed to calculate left ventricular end-diastolic volume (LVEDV), left ventricular end-systolic volume (LVESV), and left ventricular ejection fraction (LVEF). LGE was defined as an area of hyperenhancement, with higher signal intensity (≥2 SD) compared to a remote region in the same slice. Short-axis delayed enhancement images were evaluated for the presence of scars and were traced manually to measure total scar volume. Myocardial and scar volume were calculated as (Area myocardium or Area scar × slice thickness of 10 mm). The scar percentage of myocardial volume was also expressed as a percentage of the total myocardial volume (Volume scar/Volume myocardium × 100) [12].

### Statistical Methods

Data are expressed as the mean value ± SD. Comparisons between groups were performed with Student’s t, χ^2^, or Fisher’s exact tests. Comparisons between imaging parameters were made by calculating the correlation coefficient. Multivariate linear models were used to assess whether associations were maintained after adjusting for gender, medcations, and hemodynamic parameters. A p-value <0.05 was considered significant.

## Results

### Patient Characteristics

Among 47 patients, twenty-four had non-dipper BP patterns. Patients with non-dipper BP patterns and patients with dipper BP patterns were similar in terms of gender, age, body mass index, diabetes, and status of taking anti-hypertensive agents and statin (Table 1). Patients with non-dipper BP patterns had higher plasma brain natriuretic peptide (BNP) levels than patients with dipper BP patterns (152±135.7 pg/ml vs. 89.1±107.8 pg/ml, p = 0.04). There was no significant difference in renal function between non-dipper and dipper BP groups (eGFR 57.6±16.6 mL/min/m^2^ vs. 60.5±14.5 mL/min/m^2^, p = 0.264). The results of 24-h ABPM are summarized in Table 2. Though there was no significant difference in whole-day averaged systolic BP between both groups, patients with non-dipper BP patterns had higher nocturnal systolic and diastolic BP than patients with dipper BP patterns (p = 0.009 and p = 0.041).

**Table 1:** Patient characteristics.

	Non-Dipper BP	Dipper BP	P Value
	(n = 24)	(n = 23)	
Age (years)	74.3±10.1	71.5±10.7	0.181
Male	12 (50%)	17 (74%)	0.135
BMI (kg/m^2^)	25.1±3.2	24.4±3.3	0.254
Diabetes	2 (8%)	5 (22%)	0.244
Dyslipidemia	5 (21%)	6 (26%)	0.740
ACEI/ARB	19 (79%)	14 (61%)	0.212
Statin	6 (25%)	6 (26%)	1.000
β-blocker	9 (38%)	4 (17%)	0.193
Diuretics	13 (54%)	6 (26%)	0.075
BNP(pg/ml)	152±135.7	89.1±107.8	0.042

Values are expressed as the mean ± SD. BP = blood pressure; BMI = body mass index; ACEI = angiotensin-converting enzyme inhibitor; ARB = angiotensin receptor blocker; BNP = brain natriuretic peptide.

**Table 2:** 24-h ABPM.

	Non-Dipper BP	Dipper BP	P Value
	(n = 24, mm Hg)	(n = 23, mm Hg)	
Averaged SBP	135.6±15.6	137.0±13.6	0.367
Averaged DBP	78.0±7.9	77.7±11.0	0.451
Daytime SBP	136.9±16.4	143.7±14.6	0.068
Daytime DBP	79.1±8.0	81.3±11.0	0.103
Nocturnal SBP	132.8±15.8	122.6±13.0	0.009
Nocturnal DBP	75.3±9.1	69.8±11.8	0.041

Values are expressed as the mean ± SD. ABPM = ambulatory blood pressure monitoring; DBP = diastolic blood pressure; SBP = systolic blood pressure.

### CMR Parameters

**Figure 1:**
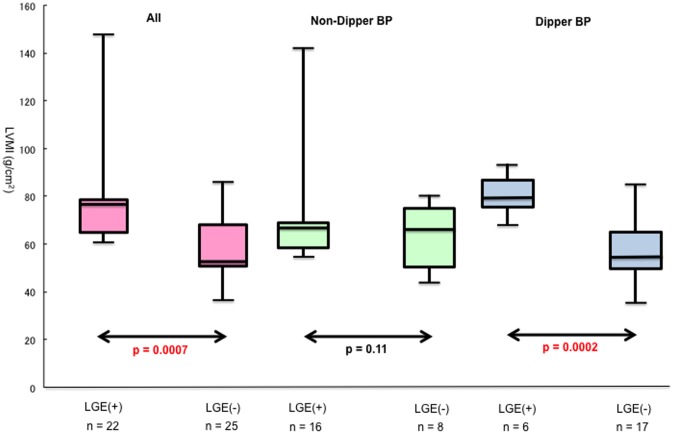
LVMI in patients with and without LGE. In 47% of patients (n = 22), LGE (late gadolinium enhancement) could be detected. In all patients and those with nocturnal dipper BP (blood pressure) patterns, LGE positive groups had larger LVMI (left ventricular mass index) than the LGE negative group (p = 0.0007 and p = 0.0002).

MRI parameters among the two patients groups are summarized in Table 3. There were no significant differences in LVEDVI, LVESVI, LVEF, and SVI between non-dipper and dipper BP groups. In contrast, patients with nocturnal non-dipper BP patterns had larger LV mass indexes (LVMI), LGE volumes, and LGE % myocardium than those of patients with dipper BP patterns (73.6±20.7 g/m^2^ vs. 63.6±15.8 g/m^2^, p = 0.035; 76.6±124.9 cm^3^ vs. 11.2±36 cm^3^, p = 0.014; 6.0±10.8% vs. 0.8±2.4%, p = 0.015; respectively). In 47% of patients (n = 22), LGE could be detected. In all patients and those with nocturnal dipper BP patterns, LGE positive groups had larger LVMI than the LGE negative group (p = 0.0007 and p = 0.0002) (Figure 1). However, there was no significant difference between groups with and without LGE in patients with nocturnal non-dipper BP patterns. In addition, logistic regression analysis (Table 4.) identified non-dipper BP patterns as independent risk factor of LGE.

**Table 3:** CMR findings.

	Non-Dipper BP	Dipper BP	P value
	(n = 24)	(n = 23)	
LVEDVI (ml/m^2^)	77.6±21.9	75.0±18.7	0.331
LVESVI (ml/m^2^)	31.8±22.0	29.3±17.3	0.335
LVEF (%)	60.9±16.5	63.1±14.0	0.309
SVI (ml/m^2^)	45.8±14.1	45.7±10.6	0.485
CI (l/min/m^2^)	3.1±0.9	2.8±0.8	0.087
LVMI (g/m^2^)	73.6±20.7	63.6±15.8	0.035
LGE (cm^3^)	76.6±124.9	11.2±36	0.014
LGE %	6.0±10.8	0.8±2.4	0.015
Myocardium (%)			

Values are expressed as the mean ± SD. CMR = cardiac magnetic resonance; LVEDVI = left ventricular end diastolic volume index; LVESVI = left ventricular end systolic volume index; LVEF = left ventricular ejection fraction; SVI = systolic volume index; CI = cardiac index; LVMI = left ventricular mass index; LGE = late gadolinium enhancement.

**Table 4:** Logistic regression analysis of the risk of LGE.

	OR (95%CI)	P value
Male	0.502 (0.085–2.980)	0.448
ACEI/ARB	2.196 (0.261–18.49)	0.469
β-blocker	0.069 (0.010–0.499)	0.008
Diuretics	0.795 (0.148–4.273)	0.789
Nocturnal systolic BP	1.016 (0.964–1.071)	0.549
Non-Dipper BP patterns	5.073 (1.046–24.61)	0.044

LGE = late gadolinium enhancement; ACEI = angiotensin-converting enzyme inhibitor; ARB = angiotensin receptor blocker; BP = blood pressure.

## Discussion

Mechanical stress by pressure overload is one of the most important factors that develops LVH. Nocturnal non-dipper BP causes contiguous pressure overload and plays an important role in target organ damage [13]. Ambulatory BP decline from day to night is associated with a lower LV mass as evaluated by an echocardiographic study in hypertensive patients [14]. The present study showed nocturnal non-dipper BP caused not only LV hypertrophy but also LGE as evaluated by comprehensive CMR for the first time. LGE can characterize the extent of interstitial fibrosis [15] that is caused by the growth of fibroblasts activated by pressure overload [16]. These comprehensive CMR findings may help to predict the precise effects of pressure overload.

### Effect of Nocturnal Non-dipper BP

In the present study, patients with nocturnal non-dipper BP patterns had higher plasma BNP levels than patients with nocturnal dipper pattern BPs. The plasma BNP level provides information on outcome and on the presence of LVH in hypertensive patients [17] [18]. Our data also showed that patients with non-dipper BP patterns had higher nocturnal systolic BP than patients with dipper BP patterns; although whole-day averaged systolic BP did not show a difference between the two groups. Nocturnal BP is superior to average and daytime BP as a predictor of cardiovascular events and all cause mortality [6] [19]. The increase in nocturnal BP is very important in patients with LVH and anti-hypertensive medication. Interestingly, in the non-dipper BP group, there was no significant difference in LVMI between LGE positive and LGE negative patients. Such a myocardium seems not to have progressed until fibrosis, but may have high-risk cardiomyocyte hypertrophy. It may be necessary to strengthen treatment even if LGE was not detected in this population group.

Among HCM patients, both the presence and extent of LGE were reported to be good independent predictors of all-cause mortality, cardiac mortality [8], and the occurrence of ventricular arrhythmia [20]. Some serum markers (ex. Procollagen type 1) have the possibility of assessing the degree of myocardial fibrosis in order to refine the ability to predict outcome in AH [21], and LGE is thought to have a similar potential.

### Study Limitations

There are several issues that should be considered when interpreting our data. The first is the selection of our study population. Because the present study was not dependent on randomized sampling, results may have been affected by selection bias. Some patients did not have LVH in cine images, though patients were recruited by echocardiographic left ventricle wall thickness. Second, because treatment for hypertension had already been provided at the time when this study was conducted, nocturnal BP may have been affected by medication for hypertension.

### Conclusion

Among hypertensive patients with LVH, the nocturnal non-dipper BP pattern was associated with both LV remodeling and myocardial degeneration independent of LV etiology, which may be associated with worsening heart failure.
